# COVID-19 Vaccination Coverage and Associated Factors Among Underserved Communities in South Carolina: Results from a Cross-Sectional Study

**DOI:** 10.1007/s40615-025-02601-8

**Published:** 2025-08-14

**Authors:** Mufaro Kanyangarara, Sachi Vora, Fatima Seck, Neal Dhankhode, Paddington T. Mundagowa

**Affiliations:** Department of Epidemiology and Biostatistics, Arnold School of Public Health, University of South Carolina, Columbia, SC USA

**Keywords:** COVID-19 vaccination, Vaccine hesitancy, African Americans, South Carolina

## Abstract

**Background:**

The effectiveness of COVID-19 vaccines in reducing adverse health outcomes is well-documented. However, vaccine uptake remains suboptimal, particularly among minority populations in the USA. We estimated COVID-19 vaccination coverage and investigated factors associated with vaccination status among adults in underserved communities in South Carolina.

**Methods:**

A cross-sectional survey was conducted among barbershop and salon clients (*n* = 330) in six counties from August to November 2023. Data were collected on demographics, health status, vaccination history for COVID-19 and the flu, concerns regarding COVID-19, and sources of health information. The likelihood of partial and full vaccination compared to no vaccination was estimated using multinomial logistic regression models.

**Results:**

About 19.4% were fully vaccinated, and 54.5% were partially vaccinated. Not receiving a flu vaccine in the past year and worrying about vaccine safety were associated with reduced likelihood of partial or full vaccination. Increased age and worry about the spread of COVID-19 were associated with an increased likelihood of vaccination. Receiving COVID-19-related information from family/friends or healthcare providers was associated with an increased likelihood of full vaccination but not partial vaccination. Religious affiliation, having no chronic health conditions, and prior COVID-19 infection were associated with a lower likelihood of partial vaccination but not full vaccination.

**Conclusion:**

The findings highlight public health concerns surrounding vaccination, the influence of religion, perceptions of invulnerability, and the role of trusted sources in vaccine decision-making. Public health messaging promoting vaccination should address these concerns and target populations often underserved by conventional healthcare systems.

## Introduction

The COVID-19 pandemic precipitated a global public health crisis. Global cumulative statistics showed that an estimated 775 million people had contracted COVID-19, and more than 7 million people had succumbed to the disease as of May 17, 2024 [1]. In the USA, the pandemic exacerbated pre-existing health disparities, disproportionately impacting racial and ethnic minorities, notably African Americans [2, 3]. African Americans were at heightened risk for COVID-19 infection and severe outcomes, and they experienced increased rates of hospitalization and mortality compared to other racial groups [3]. Despite making up only 13% of the US population, African Americans accounted for nearly 25% of COVID-19-related mortality [3], were four times more likely to be hospitalized, and had an increased risk of intensive care unit admission (relative risk = 1.93) compared to White individuals [4]. The high prevalence of underlying chronic health conditions such as hypertension, diabetes, and asthma among African Americans further amplified their vulnerability to coronavirus infection [5].

To reduce the spread of COVID-19, the federal government fast-tracked the distribution of safe and highly effective COVID-19 vaccines. Central to the versatile distribution of vaccines was ensuring universal vaccine access through coordinated programs at federal, state, tribal, and local governments, as well as public and private partners, by reaching socioeconomically, culturally, and educationally diverse populations [6]. The introduction of the COVID-19 vaccine presented an unprecedented opportunity to reduce excess mortality and eliminate disparities due to the disease. However, despite these efforts, vaccine uptake among racial and ethnic minorities was lagging. This has been attributed to different reasons, including higher rates of vaccine hesitancy, institutional mistrust, and systemic inequities in access to healthcare [7]. In addition, there was a lack of reliable information regarding the vaccine, complacency, and structural barriers to vaccine access among ethnic minority groups [8]. Thus, there was a need for locally acceptable approaches and institutions to improve vaccine uptake and health prognosis for the minority populations in the USA.

Several efforts to improve COVID-19 vaccination rates among high-risk, underserved populations leveraged community institutions such as workplaces, places of worship, barbershops, and hair salons. Local communities often trust the patrons of these places to help with information dissemination and provide space for testing, contact tracing, and vaccination [9]. Historically, barbershops and salons have been important locations for political activism, socialization, and solace where community members and patrons openly discuss matters affecting their community without fear of prejudice [10]. Understanding the characteristics of the people who utilize barbershops and salons may help inform interventions that can be implemented through these important institutions, particularly in states with wide COVID-19 service access gaps, such as South Carolina.

In 2024, 26.2% of South Carolina’s population identified as Black/African American [11] and COVID-19-related morbidity and mortality statistics revealed that African American people were 8.4% more likely to get a COVID-19 test, 15.1% more likely to test positive for COVID-19, and had a higher COVID-19-related death rate compared to White people [12]. Effective April 13, 2024, hospitals were no longer mandated to report Coronavirus cases, and by that time, South Carolina had reported 1,857,853 COVID-19 cases and 20,311 deaths [13]. Approximately 59.9% of the South Carolina population was fully vaccinated, and the state was ranked 37th out of 51 states in terms of the population fully vaccinated [14]. Barbershops and salons are easy entry points to minority communities and can help to provide a lens into the nuanced health characteristics such as knowledge and attitudes as well as cues on possible strategies to intervene. Understanding the drivers of vaccination among the underserved in South Carolina can support the development of targeted interventions to increase vaccine uptake and reduce health disparities. This study aimed to assess COVID-19 vaccination coverage and associated factors among adults living in underserved communities in South Carolina. This study sought to provide evidence on how barbershops and salons can be leveraged to improve our responsiveness to future public health responses by utilizing the shared community values, relationships, and culture between the barbers and stylists with the community.

## Methods

### Study Design and Participants

This cross-sectional study was conducted as part of a collaborative effort between the University of South Carolina (USC), the South Carolina Department of Health and Environmental Control (SCDHEC), and community partners under the national initiative to address COVID-19 health disparities among populations at high risk and underserved, including racial and ethnic minority populations and rural communities. Surveys were administered across six counties: Darlington (PeeDee region), Lee (Pee Dee region), Lexington (Midlands region), Orangeburg (Low Country region), Richland (Midlands region), and Sumter (Midlands region). These counties were purposively selected due to their sizable African American and other ethnic minority populations and lower COVID-19 testing and vaccination rates among the underserved, particularly among African Americans.

Barbershops and salons provide supportive spaces where clients build trusted relationships with providers and openly discuss health and personal issues. A list of potential barbershops and salons was created based on a compilation of licensed establishments from the South Carolina Department of Labor, Licensing and Regulation, searches on online marketplaces such as StyleSeat and The Cut, and referrals from community partners. Geographical locations were narrowed to zip codes predominantly inhabited by African Americans.

Surveys were administered to clients in barbershops and salons within the selected counties, as these venues are significant sociocultural institutions in underserved neighborhoods and minority communities in the USA. Using a recruitment script, owners of prospective barbershops and salons were contacted to determine interest and obtain permission to survey their clients. In-person visits were scheduled for the interested venues, typically occurring on Thursdays through Saturdays in the afternoons, which were the peak business hours. Convenience sampling was used to approach potential participants after they had received the intended services. The inclusion criteria required participants to be 18 years or older, willing and able to provide consent, and a client of the barbershop or salon.

### Data Collection

After providing informed consent, participants completed a self-administered questionnaire on an Android tablet provided by a research assistant or by scanning a QR code to access it on their personal mobile phones. Research assistants were available on-site to assist participants with limited technological literacy. The structured questionnaire collected data on sociodemographic characteristics (e.g., age, gender, marital status, and level of education), health status (e.g., pre-existing medical conditions), healthcare access (e.g., recent flu vaccination), health insurance coverage, and COVID-19-related knowledge, attitudes, perceptions, vaccination status, and testing history. Questions were adapted from previously used data collection tools [15], including the Kaiser Family Foundation COVID-19 Vaccine Monitor Survey [16] and the Centers for Disease Control and Prevention (CDC) COVID-19 Community Survey Question Bank [17].

Before data collection, the survey was pilot tested with 10 participants to ensure functionality, feasibility, and comprehensibility; these participants were excluded from the final dataset. Data were collected using Open Data Kit (ODK) software on password-protected tablets and uploaded to a secure server. The survey incorporated field validation checks, branching logic, and skip patterns to reduce data entry errors and missing data. The survey was conducted between August and November 2023, taking approximately 20 min to complete. Participants received a $25 Visa gift card for their time and participation. A total of 349 respondents completed the survey.

### Measures

The outcome variable, vaccination status, was self-reported and categorized into three levels: unvaccinated, partially vaccinated, and fully vaccinated. The study outcome was based on responses to the survey question, “Did you get a COVID-19 vaccine?”, with responses recorded as Yes (partially vaccinated with one dose), Yes (fully vaccinated with two doses), Yes (fully vaccinated with two doses plus the booster), and No. Unvaccinated was used as the reference category, signifying non-adherence to vaccination guidelines. Individuals who refused to report their vaccination status were excluded from the analysis.

Covariates were selected based on previous studies and our research objectives. The sociodemographic information collected included age, gender, race, marital status, education, employment status, household size, religion, and household income. Age was categorized into three groups: 18–34 years, 35–49 years, and 50 years or older. Gender was dichotomized as male or non-male, which included females and participants who identified as non-binary/third gender, transgender, and other gender. Dichotomization of male or non-male was selected due to small numbers of non-binary or gender-diverse participants (less than 1%). Self-identified race was grouped into Black/African American or non-Black/African American. Marital status was categorized into never married or other (i.e., married, divorced, widowed, or separated). The highest level of education completed was dichotomized as either having less than a college education or having some college education/higher. Employment status was coded as employed full-time or not (i.e., employed part-time, homemaker, student, retiree, or unable to work). Household size was based on the number of family members currently residing with the participant, distinguishing between households with less than three family members and those with three or more members. Annual household income was dichotomized as less than $30,000, $30,000 and above, or do not know/refused. For religion, participants were asked the question: “Do you belong to any particular religion?” with Yes, No, and Prefer not to say as the possible responses. Healthcare access variables included the number of physician visits in the last 6 months (dichotomized as one or more vs none), health insurance coverage (yes vs no), and receipt of a flu vaccine in the last 12 months (yes vs no). Participants self-reported the presence of chronic conditions including hypertension, diabetes, asthma, and obesity (yes vs no).

### Statistical Analysis

Descriptive statistics were calculated to summarize the sociodemographic and health characteristics of the sample, as well as knowledge, attitudes, and practices related to COVID-19 testing and vaccination. Chi-square tests were used to assess significant differences in these characteristics by vaccination status. To examine associations of risk factors with COVID-19 vaccination status, multinomial logistic regression was employed, using the unvaccinated group as the reference category. Although the outcome, vaccination status could conceptually be considered as ordinal, we used multinomial logistic regression rather than ordinal logistic regression because the proportional odd assumption was not met, based on the Brant test. First, unadjusted multinomial logistic regression models were fit for each independent variable against the polytomous vaccination status variable. Variables that were statistically significant (*p* ≤ 0.05) or that resulted in a change in parameter estimate of at least 20% were considered for inclusion in the adjusted multinomial logistic regression model. Age was included in the model a priori. Model fit was assessed using the generalized Hosmer–Lemeshow goodness-of-fit test for multinomial logistic regression models. Multicollinearity was assessed by examining the conditioning indices, with a condition number of 30 or greater used as a cut-off value. Relative risk ratios (RRRs) and 95% confidence intervals (CIs) were reported for all variables in the adjusted multinomial logistic regression model. A complete case analysis was used since there were no missing data. We conducted sensitivity analyses by re-running the multinomial logistic regression after excluding individuals who selected “don’t know” or “refused” for key covariates (e.g., income), and also using ordinal logistic regression. Results from the sensitivity analyses were generally consistent with the primary findings in terms of the direction and magnitude of associations.

All statistical analyses were performed using STATA SE version 17.0. A *p* value of < 0.05 was considered statistically significant. Findings were reported in accordance with the Strengthening the Reporting of Observational Studies in Epidemiology (STROBE) reporting guidelines.

### Ethical Considerations

The University of South Carolina Institutional Review Board deemed this study exempt from review (date July 7, 2023, Number Pro00129899). Barbershops and salon owners were asked for permission to administer the questionnaire to clients at their establishments. Participation was voluntary, and all participants provided informed consent after being fully briefed on the study’s purpose, potential risks, and their rights, including the right to withdraw at any time.

## Results

### Participant Characteristics

Of the 349 study participants, 19 were excluded due to missing data on the outcome, resulting in a final sample size of 330. The population was predominantly male (57.6%), Black/African American (93.0%), less than 50 years old (70.6%), and never married (52.1%), with 57.9% employed full-time (Table 1). Half of the participants had some college education or higher (50%). In terms of healthcare access, the majority had at least one physician visit in the previous 6 months (82.7%) and did not receive a flu vaccine in the previous 12 months (71.2%). The most common health conditions were hypertension (23.6%), diabetes (12.4%), asthma (9.7%), and obesity (6.1%).

**Table 1:** Characteristics of the study participants by vaccination status

Characteristic	Total (*n* = 330) *n* (%)	Fully Vaccinated (*n* = 64) *n* (%)	Partially Vaccinated (*n* = 180) *n* (%)	Unvaccinated (*n* = 86) *n* (%)	*p*-value
Age range					< 0.01
18–34 years	124 (37.6)	14 (21.9)	58 (32.2)	52 (60.5)	
35–49 years	109 (33.0)	12 (18.8)	72 (40.0)	25 (29.1)	
50 + years	97 (29.4)	38 (59.4)	50 (27.8)	9 (10.5)	
Male	194 (58.8)	26 (40.6)	112 (62.2)	56 (65.1)	< 0.01
Black/African American	307 (93.0)	61 (95.3)	166 (92.2)	80 (93.0)	0.70
Attended at least some college or higher	165 (50.0)	38 (59.4)	96 (53.3)	31 (36.0)	< 0.01
Never married	172 (52.1)	22 (34.4)	85 (47.2)	65 (75.6)	< 0.01
Belongs to a particular religion	104 (31.5)	29 (45.3)	63 (35.0)	12 (14.0)	< 0.01
Employed full-time	191 (57.9)	26 (40.6)	112 (62.2)	53 (61.6)	< 0.01
Less than 3 household members	142 (43.0)	36 (56.3)	76 (42.2)	30 (34.9)	0.03
Household income less than $30,000	96 (29.1)	20 (31.3)	46 (25.6)	30 (34.9)	0.60
One or more physician visits in the last 6 months	273 (82.7)	57 (89.1)	154 (85.6)	62 (72.1)	< 0.01
Covered by health insurance	273 (82.7)	59 (92.2)	154 (85.6)	60 (69.8)	< 0.01
Had hypertension	78 (23.6)	23 (35.9)	47 (26.1)	8 (9.3)	< 0.01
Had diabetes	41 (12.4)	10 (15.6)	27 (15.0)	4 (4.7)	0.04
Had asthma	32 (9.7)	5 (7.8)	20 (11.1)	7 (8.1)	0.60
Were obese	20 (6.1)	8 (12.5)	11 (6.1)	1 (1.2)	0.02
Did not receive flu vaccine in the last 12 months	235 (71.2)	26 (40.6)	131 (72.8)	78 (90.7)	< 0.01

Based on self-reported vaccination status, 19.4% of participants were fully vaccinated, 54.5% were partially vaccinated, and 26.1% were unvaccinated (Table 1). Significant differences in sociodemographic characteristics were observed by vaccination status. Most fully vaccinated individuals were aged 50 or older (59.4%), while the unvaccinated individuals were predominantly younger (18–34 years 60.5%). Females were more likely to be fully vaccinated (59.4%), while males were more likely to be partially vaccinated (62.2%) or unvaccinated (65.1%). A higher percentage of fully vaccinated individuals had some college education or higher (59.4%) compared to unvaccinated individuals (36.0%). Fully vaccinated individuals were less likely to be fully employed (40.6%) compared to the partially vaccinated (62.2%) and unvaccinated (61.6%). The fully vaccinated (45.3%) were more likely to belong to a religion compared to the unvaccinated (14.0%).

There were also differences in health status and healthcare access by vaccination status (Table 1). Chronic health conditions (hypertension, diabetes, and obesity) were more common among the fully vaccinated (hypertension 35.9%, diabetes 15.6%, obesity 12.5%) than the unvaccinated (hypertension 9.3%, diabetes 4.7%, obesity 1.2%). Fully vaccinated individuals had one or more physician visits recently (89.1%) compared to the unvaccinated (72.1%). Moreover, health insurance coverage was significantly higher among the fully vaccinated (92.2%) compared to the unvaccinated (69.8%).

### Experiences and Attitudes Related to COVID-19

**Fig. 1:**
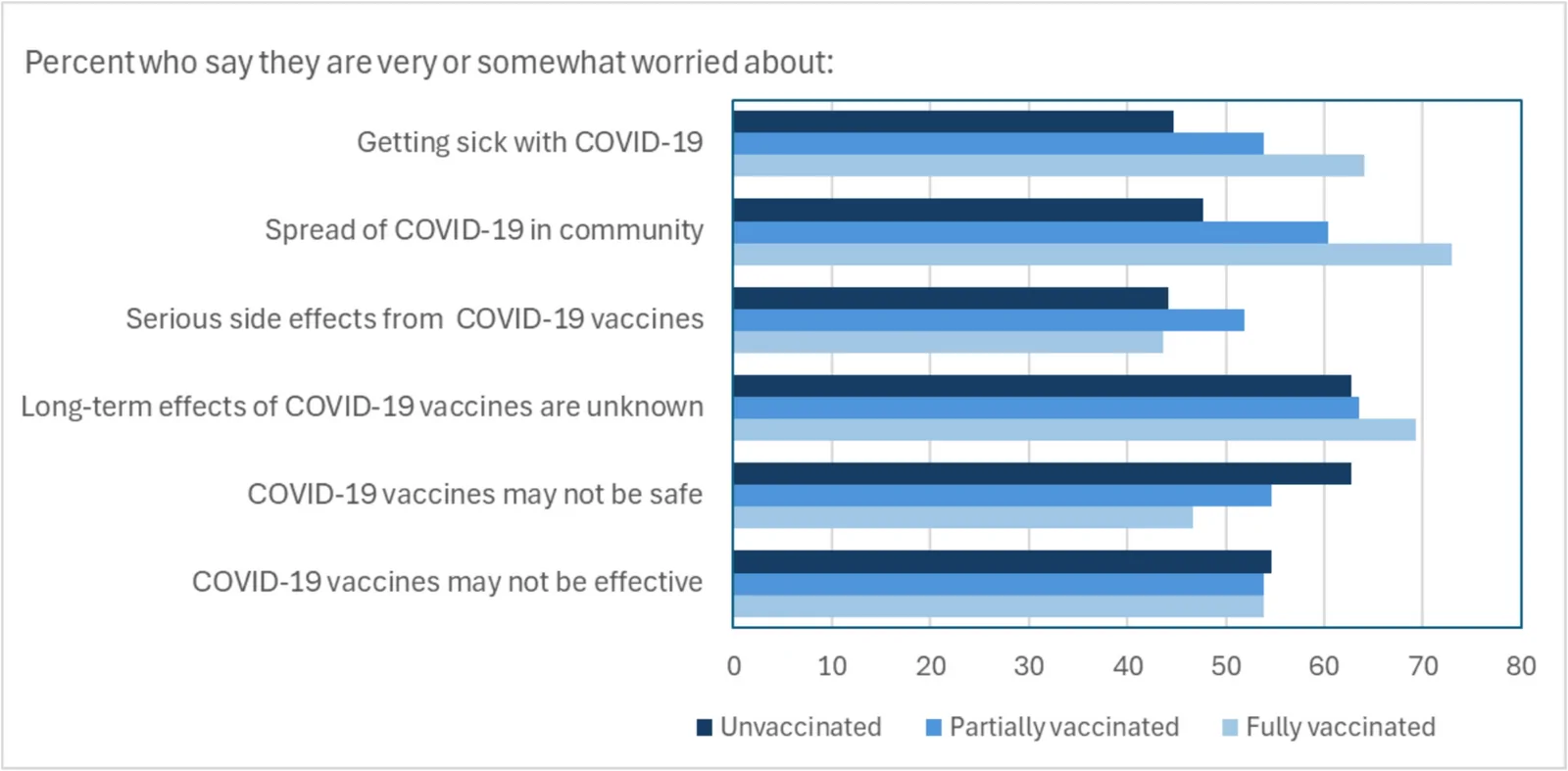
Perceived threat of COVID-19 and attitudes towards COVID-19 vaccines

Participants expressed varying levels of concern about contracting COVID-19, the community spread of COVID-19, the serious side effects of COVID-19 vaccines, and the safety, effectiveness, and long-term effects of COVID-19 vaccines (Fig. 1). Compared to partially vaccinated and unvaccinated individuals, fully vaccinated individuals were more worried about the spread of COVID-19 in their community (73.0%), getting sick with COVID-19 (64.1%), and the unknown long-term effects of COVID-19 vaccines (69.3%). In contrast, partially vaccinated individuals were more worried about experiencing serious side effects from COVID-19 vaccines (51.9%) compared to fully vaccinated (43.7%) and unvaccinated individuals (44.2%). Unvaccinated individuals had the highest concerns about the safety of COVID-19 vaccines (62.8%) compared to fully vaccinated (46.7%) and partially vaccinated individuals (54.7%). Notably, concerns about the effectiveness of COVID-19 vaccines were relatively similar across the groups (range 53.9–54.6%).

### Factors Associated with Vaccination

Table 2 shows the results of the adjusted multinomial logistic regression analyses, using the unvaccinated group as the reference category, and displaying significant associations with the likelihood of partial or full vaccination. Individuals aged 35–49 years were significantly more likely to be partially vaccinated (RRR = 2.44, 95% CI 1.24–4.80) compared to those aged 18–34 years, while those aged 50 years and above were more likely to be both partially (RRR = 2.85, 95% CI 1.15–7.07) and fully vaccinated (RRR = 8.58, 95% CI 2.63–27.96). Gender did not have a statistically significant effect on vaccination status (partially vaccinated RRR = 1.57, 95% CI 0.80–3.06; fully vaccinated RRR = 1.11, 95% CI 0.45–2.74). Religious affiliation was associated with a lower likelihood of partial vaccination (RRR = 0.42, 95% CI 0.19–0.94); however, the association was not significant for full vaccination (RRR = 0.49; 95% CI 0.18–1.36).

**Table 2:** Results of the adjusted multinomial logistic regression analyses (ref: unvaccinated)

Characteristic	Partially vaccinated RRR (95% CI)	Fully vaccinated RRR (95% CI)
Age range		
18–34 years	Reference	Reference
35–49 years	2.44 (1.24–4.80)*	1.99 (0.68–5.86)
50 + years	2.85 (1.15–7.07)*	8.58 (2.63–27.96)*
Male	1.57 (0.80–3.06)	1.11 (0.45–2.74)
Belongs to a particular religion	0.42 (0.19–0.94)*	0.49 (0.18–1.36)
No chronic health problems	0.34 (0.16–0.74)*	0.48 (0.17–1.33)
Did not receive flu vaccine in the last 12 months	0.23 (0.09–0.59)*	0.07 (0.02–0.20)*
Worried COVID-19 vaccines may not be safe	0.29 (0.14–0.61)*	0.12 (0.05–0.34)*
Worried about the spread of COVID-19 in the community	2.23 (1.10–4.49)*	7.11 (2.49–20.31)*
Source of COVID-19 information: family/friends	1.74 (0.85–3.56)	2.87 (1.13–7.27)*
Source of COVID-19 information: healthcare provider	1.79 (0.84–3.81)	3.74 (1.42–9.85)*
Never had COVID-19	0.34 (0.19–0.65)*	0.42 (0.17–1.01)

*CI* confidence interval, *RRR* relative risk ratio

*p < 0.05

Individuals without health problems were less likely to be partially vaccinated than those with chronic health problems (RRR = 0.34, 95% CI 0.16–0.74); however, the effect of health problems on full vaccination was not significant. Those who did not receive the flu vaccine in the last 12 months were significantly less likely to be both partially (RRR = 0.23, 95% CI 0.09–0.59) and fully vaccinated (RRR = 0.07, 95% CI 0.02–0.20). Concern about COVID-19 vaccine safety was associated with a significant reduction in both the likelihood of partial (RRR = 0.29, 95% CI 0.14–0.61) and full vaccination (RRR = 0.12, 95% CI 0.05–0.34). Conversely, worry about the spread of COVID-19 in the community increased the likelihood of both partial (RRR = 2.23, 95% CI 1.10–4.49) and full vaccination (RRR = 7.11, 95% CI 2.49–20.31). Relying on family or friends (RRR = 2.87, 95% CI 1.13–7.27) and healthcare providers (RRR = 3.74; 95% CI 1.42–9.85) as a source of COVID-19 information significantly increased the likelihood of full vaccination. Individuals who reported never contracting COVID-19 were less likely to be partially vaccinated (RRR = 0.34, 95% CI 0.19–0.65); nonetheless, the association with full vaccination was not significant (RRR = 0.42; 95% CI 0.17–1.01).

## Discussion

South Carolina consistently ranked among the states with the lowest COVID-19 vaccination coverage. This study aimed to assess COVID-19 vaccination coverage and factors associated with vaccination status among adults in underserved communities in South Carolina. Our findings indicated that about one in five adults were fully vaccinated, with a little over half partially vaccinated. This was inconsistent with state-level data showing that 54.3% of eligible residents were fully vaccinated [18] and 71% had received at least one dose [19]. The findings highlight the significant discrepancy between COVID-19 vaccination rates in underserved areas and overall state-level data. Underserved communities often face barriers to accessing healthcare services, including vaccination sites, mainly due to a lack of nearby facilities, transportation issues, or limited availability of appointments [20]. Socioeconomic challenges such as taking time off work [20], misinformation, and hesitancy may influence vaccine coverage in underserved communities. In addition, state-level data might reflect broader outreach efforts that were not as effective in reaching underserved populations. Tailored communication and community engagement were crucial for improving vaccination rates in these areas [21].

Age significantly influenced vaccination status in this study, with older adults 50 and older being significantly more likely to be partially and fully vaccinated than younger adults (18–34 years). This trend mirrors national data, which shows higher vaccination rates among older populations, likely due to their increased risk of severe COVID-19 outcomes and heightened awareness of age-related health risks [22]. Additionally, adults without chronic health conditions were less likely to be partially vaccinated, potentially due to a reduced risk perception of vulnerability to infection and severe illness. This trend was consistent with other studies on COVID-19 vaccination behavior, which revealed that individuals without chronic conditions often perceive their risk of severe COVID-19 as lower, contributing to lower vaccination rates [23, 24]. The perception of invulnerability among these groups suggests that public health campaigns should emphasize vaccination benefits for all, irrespective of age or current health status. Highlighting the role of vaccines in protecting both personal and community health could resonate with and motivate younger individuals and those without chronic health conditions who may otherwise feel invulnerable to severe COVID-19 impacts.

Individuals who had not received a flu vaccine in the previous year were less likely to be partially or fully vaccinated against COVID-19. This is consistent with findings from similar studies, which report that individuals who are resistant to vaccines in general are often less motivated to get vaccinated against COVID-19, believing they are at lower risk of severe outcomes [25, 26]. Promoting a holistic perspective on vaccination, including annual flu shots, could improve vaccination uptake for COVID-19 and other vaccine-preventable diseases [27].

Our study also highlighted the influence of religion on vaccination behavior, as individuals with a religious affiliation had a reduced likelihood of being partially vaccinated. Previous studies show that faith-based perspectives can shape health behaviors such as vaccine decision-making. Religious individuals tend to rely on their faith in divine protection rather than medical intervention [28–33]. Given that places of worship hold significant influence within African American communities, involving faith leaders and faith-based organizations in vaccine outreach efforts and developing tailored, respectful communication that addresses religious beliefs alongside evidence-based vaccine information could be effective in reducing vaccine hesitancy [3, 34].

Concerns about vaccine safety and effectiveness were strong deterrents to vaccination, as evidenced by a reduced likelihood of both partial and full vaccination among individuals worried about the safety of COVID-19 vaccines. These concerns are consistent with other studies that have shown similar apprehensions among vaccinated individuals [35, 36]. The significant concern about vaccine safety among unvaccinated individuals also suggests that messaging should provide clear, evidence-based information about vaccine safety and efficacy. On the other hand, concerns about the spread of COVID-19 in the community and concerns about acquiring COVID-19 were proponents of vaccination, as individuals who were fully vaccinated tended to worry about the spread of COVID-19 in the community, acquiring COVID-19 and the long-term effects of COVID-19 evidenced by an increased likelihood of both partial and full vaccination among individuals. Given the worries expressed by vaccinated individuals, integrating mental health support and providing ongoing public health messaging to reassure vaccinated individuals about vaccine efficacy in preventing severe outcomes and reducing transmission could help sustain trust in future public health campaigns.

Lastly, we found that individuals who sought COVID-19-related information from family, friends, and healthcare providers were more likely to be fully vaccinated. This finding is consistent with previous research that indicates that trusted sources of information play a role in shaping vaccine acceptance, intentions, and behaviors [37]. For instance, conversations among family members and friends, particularly for African American women, have been linked to intentions to adopt protective health behaviors [38]. The observed role of healthcare providers and social networks in vaccination decisions highlights the potential of leveraging these trusted relationships to disseminate accurate, reassuring information about COVID-19 vaccines [39]. Public health strategies should empower healthcare providers and community leaders to disseminate accurate and reassuring information about COVID-19 vaccines.

Several limitations should be considered in the interpretation of these findings. First, the COVID-19 coverage observed in this study is based on adults from underserved areas in South Carolina, which may limit generalizability to all population groups, as vaccine hesitancy can vary based on specific community and contextual factors. Given that the majority of our participants were African Americans, future efforts should also consider evaluating whether racial concordance existed with healthcare providers, and if that contributed to increased vaccinations. Second, the use of a convenience sample of adults visiting barbershops and salons could introduce selection bias and influence the study’s findings. Third, the cross-sectional design limits the ability to make causal inferences. Lastly, we could not verify participants’ vaccination status through state records or health provider reports, and the use of self-reported data may be subject to social desirability bias. Limitations notwithstanding, this study identifies factors influencing COVID-19 vaccination and provides recommendations to improve vaccine uptake in underserved settings in South Carolina. While the immediate threat of COVID-19 has declined, our findings serve as a valuable roadmap for informing future public health responses to emerging infectious diseases and vaccine distribution strategies.

## Conclusion

A multifaceted approach that leverages community networks and culturally relevant messaging is needed to improve COVID-19 vaccination and map the future of other vaccination programs. Collaborating with trusted local figures such as community leaders, barbers, stylists, and religious leaders can improve the reach and credibility of public health information to populations often underserved and distrustful of conventional health systems. Community-based settings like barbershops, salons, and churches, where trusted relationships are established, offer an opportunity to address vaccine hesitancy, provide accurate information, and promote health equity. Targeted messaging should acknowledge religious beliefs and address specific COVID-19 and vaccine concerns to reinforce the role of vaccines in mitigating adverse health outcomes. Messaging should target younger and healthier individuals who may underestimate their susceptibility to the severe effects of COVID-19, highlighting that COVID-19 vaccines protect individuals, and community health may encourage a more collective approach. Finally, social circles and healthcare providers often serve as a source of health information; empowering them to provide evidence-based information will allow individuals to make more informed vaccination decisions. Future efforts should consider building on these trusted community relationships to not only improve COVID-19 vaccination rates but also to address broader health disparities within African American communities. Given that our study primarily focused on African Americans, it will also be imperative to evaluate if racial concordance exists with the healthcare providers and if that contributed to increased vaccinations. While the urgency of the pandemic has diminished, our findings offer important reflections to guide future public health strategies and strengthen responses to future health crises.

## Data Availability

The data related to this study is available from the corresponding author upon a reasonable request.

## References

[1] World Health Organization (WHO). COVID-19 epidemiological update – 17 May 2024. World Health Organization. 2024. Available from: https://www.who.int/publications/m/item/covid-19-epidemiological-update-edition-167. Accessed 20 Oct 2024.

[2] Phillips N, Park I-W, Robinson JR, Jones HP. The perfect storm: COVID-19 health disparities in US Blacks. J Racial Ethn Health Disparities. 2021;8(5):1153–60. 10.1007/s40615-020-00871-y PMC7510013 32965660

[3] Centers for Disease Control and Prevention. COVID-19 provisional counts - health disparities. National Center for Health Statistics. 2023. https://www.cdc.gov/nchs/nvss/vsrr/covid19/health_disparities.htm#print. Accessed 20 Oct 2024.

[4] Magesh S, John D, Li WT, Li Y, Mattingly-App A, Jain S, et al. Disparities in COVID-19 outcomes by race, ethnicity, and socioeconomic status: a systematic-review and meta-analysis. JAMA Netw Open. 2021;4(11): e2134147. 10.1001/jamanetworkopen.2021.34147 PMC8586903 34762110

[5] Vasquez RM. The disproportional impact of COVID-19 on African Americans. Health Hum Rights. 2020;22(2):299–307. PMC7762908 33390715

[6] National Academies of Sciences, Engineering and Medicine; Board on Population Health and Public Health Practice; Board on Health Science Policy; Committee of Equitable Allocation of Vaccine for the Novel Coronavirus. A framework of equitable allocation of COVID-19 vaccine. In: Kahn B, Brown L, Foege W, editors. Allocation of Vaccine for the Novel Coronavirus. Washington (DC): National Academies Press (US); 2020. https://www.ncbi.nlm.nih.gov/books/NBK564091. Accessed 20 Oct 2024. 33026758

[7] Majee W, Anakwe A, Onyeaka K, Harvey IS. The past is so present: understanding COVID-19 vaccine hesitancy among African American adults using qualitative data. J Racial Ethn Health Disparities. 2023;10(1):462–74. 10.1007/s40615-022-01236-3 PMC8857529 35182372

[8] Shearn C, Krockow EM. Reasons for COVID-19 vaccine hesitancy in ethnic minority groups: a systematic review and thematic synthesis of initial attitudes in qualitative research. SSM. 2023;3: 100210. 10.1016/j.ssmqr.2022.100210 PMC9771578 36573229

[9] Balls-Berry J, Dacy LC, Balls J. “Heard it through the grapevine”: The Black Barbershop as a source of health information. Hektoen Int a J Med Humanit. 2015 Summer;7(3). http://hektoeninternational.org/index.php?option=com_content&view=article&id=1871. Accessed 20 Oct 2024. PMC4749262 26877889

[10] Mbilishaka A. Black lives (and stories) matter: race narrative therapy in Black hair care spaces. Community Psychol Glob Perspect. 2018;4(2):22–33.

[11] McAllister R. South Carolina Population 2025 – by age, county, city and race. North American Community Hub Statistics. 2025. https://nchstats.com/south-carolina-population/. Accessed 20 Oct 2024.

[12] South Carolina Department of Health and Environmental Control. COVID-19 and racial disparity in South Carolina. Columbia, SC; 2022.

[13] Worldometer. South Carolina. 2024. https://www.worldometers.info/coronavirus/usa/south-carolina/. Accessed 20 Oct 2024.

[14] Becker’s Hospital Review. States ranked by percentage of population fully vaccinated. 2023. https://www.beckershospitalreview.com/quality/public-health/states-ranked-by-percentage-of-population-vaccinated-march-15/. Accessed 20 Oct 2024

[15] Krzyzanowski MC, Terry I, Williams D, West P, Gridley LN, Hamilton CM. The PhenX toolkit: establishing standard measures for COVID-19 research. Current Protocols. 2021;1(4): e111. 10.1002/cpz1.111 PMC8206667 33905618

[16] Kaiser Family Foundation. KFF COVID-19 vaccine monitor archives. KFF. 2023. https://www.kff.org/coronavirus-covid-19/kff-covid-19-vaccine-monitor-archives/. Accessed 20 Oct 2024.

[17] NIH Common Data Elements (CDE) Repository. CDC COVID-19 community survey question bank. 2021. https://www.phenxtoolkit.org/toolkit_content/PDF/CDC_Community.pdf. Accessed 20 Oct 2024.

[18] South Carolina Department of Public Health. COVID-19 vaccine data. Columbia, SC; 2024. https://dph.sc.gov/diseases-conditions/infectious-diseases/covid-19/covid-19-vaccine-data. Accessed 20 Oct 2024.

[19] USA FACTS. South Carolina vaccination progress. 2023. https://usafacts.org/visualizations/covid-vaccine-tracker-states/state/south-carolina/. Accessed 20 Oct 2024.

[20] South Carolina Department of Health and Environmental Control. South Carolina congregate and underserved populations COVID-19 vaccination plan (CUPP)- Appendix B: COVID-19 Vaccine Plan. Columbia, SC; 2021. Ahttps://scdhec.gov/sites/default/files/media/document/CUPP-Vaccination-Plan-5-10-21.pdf. Accessed 20 Oct 2024.

[21] South Carolina Department of Health. DHEC, Partners work to increase access to COVID-19 vaccines in rural communities. Columbia, SC; 2021. https://scdhec.gov/news-releases/dhec-partners-work-increase-access-covid-19-vaccines-rural-communities. Accessed 20 Oct 2024.

[22] Ezeamii VC, Ofochukwu VC, Iheagwara C, Asibu T, Ayo-Farai O, Gebeyehu YH, Kaglo EO, Odoeke MC, Adeyemi OM, Shittu HO, Okobi OE. COVID-19 vaccination rates and predictors of uptake among adults with coronary heart disease: insight from the 2022 National Health Interview Survey. Cureus. 2024;16(1): e52480. 10.7759/cureus.52480. PMC10873900 38371067

[23] Schoeni RF, Wiemers EE, Seltzer JA, Langa KM. Association between risk factors for complications from COVID-19, perceived chances of infection and complications, and protective behavior in the US. JAMA Netw Open. 2021;4(3): e213984. 10.1001/jamanetworkopen.2021.3984 PMC8013830 33787906

[24] Kompaniyets L, Pennington AF, Goodman AB, Rosenblum HG, Belay B, Ko JY, et al. Underlying medical conditions and severe illness among 540,667 adults hospitalized with COVID-19, March 2020–March 2021. Prev Chronic Dis. 2021;18: 210123. 10.5888/pcd18.210123 PMC8269743 34197283

[25] Steele MK, Couture A, Reed C, Iuliano D, Whitaker M, Fast H, et al. Estimated number of COVID-19 infections, hospitalizations, and deaths prevented among vaccinated persons in the US, December 2020 to September 2021. JAMA Netw Open. 2022;5(7): e2220385. 10.1001/jamanetworkopen.2022.20385 PMC9260489 35793085

[26] Xu S, Huang R, Sy LS, Glenn SC, Ryan DS, Morrissette K, et al. COVID-19 vaccination and non–COVID-19 mortality risk — seven integrated health care organizations, United States, December 14, 2020–July 31, 2021. MMWR Morb Mortal Wkly Rep. 2021;70(43):1520–4. 10.15585/mmwr.mm7043e2 PMC8553028 34710075

[27] Du H, Saiyed S, Gardner LM. Association between vaccination rates and COVID-19 health outcomes in the United States: a population-level statistical analysis. BMC Public Health. 2024;24(1): 220. 10.1186/s12889-024-17790-w PMC10797940 38238709

[28] Chakhunashvili K, Kvirkvelia E, Chakhunashvili DG. Religious belongings and COVID-19 vaccination. BMC Public Health. 2024;24(1): 2585. 10.1186/s12889-024-20133-4 PMC11428539 39334217

[29] Chan E. Are the religious suspicious of science? Investigating religiosity, religious context, and orientations towards science. Public Underst Sci. 2018;27(8):967–84. 10.1177/0963662518781231 29874969

[30] Martens JP, Rutjens BT. Spirituality and religiosity contribute to ongoing COVID-19 vaccination rates: comparing 195 regions around the world. Vaccine X. 2022;12: 100241. 10.1016/j.jvacx.2022.100241 PMC9666266 36407820

[31] Maciuszek J, Polak M, Stasiuk K, Rosiński J. Declared intention to vaccinate against COVID-19 and actual vaccination—the role of trust in science, conspiratorial thinking and religiosity. Vaccines. 2023;11(2): 262. 10.3390/vaccines11020262 PMC9965173 36851140

[32] Olivera-Figueroa LA, Unger A, Papastamatelou J, Zimbardo PG. A time to get vaccinated? The role of time perspective, consideration of future consequences, conspiracy beliefs, religious faith, gender, and race on intention to vaccinate for COVID-19 in the United States. Int J Environ Res Public Health. 2023;20(4): 3625. 10.3390/ijerph20043625 PMC9963941 36834320

[33] Tiwana MH, Smith J. Faith and vaccination: a scoping review of the relationships between religious beliefs and vaccine hesitancy. BMC Public Health. 2024;24(1): 1806. 10.1186/s12889-024-18873-4 PMC11227154 38971784

[34] The Conversation. COVID has highlighted the connection between spirituality and vaccine skepticism. 2023. https://theconversation.com/covid-has-highlighted-the-connection-between-spirituality-and-vaccine-scepticism-203941. Accessed 20 Oct 2024.

[35] Graña C, Ghosn L, Evrenoglou T, Jarde A, Minozzi S, Bergman H, et al. Efficacy and safety of COVID-19 vaccines. Cochrane Database Syst Rev. 2022;12(12):CD015477. 10.1002/14651858.CD015477. PMC9726273 36473651

[36] University of Oxford. Ground-breaking study reveals how COVID-19 vaccines prevent severe disease. 2024. https://www.ox.ac.uk/news/2024-05-03-ground-breaking-study-reveals-how-covid-19-vaccines-prevent-severe-disease. Accessed 20 Oct 2024.

[37] Rabin Y, Kohler RE. COVID-19 vaccination messengers, communication channels, and messages trusted among black communities in the USA: a review. J Racial Ethn Health Disparities. 2023. 10.1007/s40615-023-01858-1. PMC11345940 37947953

[38] Francis DB, Zelaya CM, Fortune DA, Noar SM. Black college women’s interpersonal communication in response to a sexual health intervention: a mixed methods study. Health Commun. 2021;36(2):217–25. 10.1080/10410236.2019.1673949 PMC7138716 31590577

[39] Mundagowa PT, Vora S, Seck F, Dhankhode N, Sakyi KS, Kanyangarara M. Exploring the views of barbers and stylists on the acceptability of delivering community-based interventions to promote COVID-19 testing and vaccination in South Carolina. Vaccines. 2024;12(9):1011. 10.3390/vaccines12091011 PMC11436097 39340041

